# The influence of different deep-sea coral habitats on sediment macrofaunal community structure and function

**DOI:** 10.7717/peerj.5276

**Published:** 2018-07-20

**Authors:** Jill R. Bourque, Amanda W.J. Demopoulos

**Affiliations:** Wetland and Aquatic Research Center, US Geological Survey, Gainesville, FL, United States of America

**Keywords:** *Lophelia pertusa*, *Madrepora oculata*, Octocorals, Sediment macrofauna, Community ecology, Functional traits, Gulf of Mexico

## Abstract

Deep-sea corals can create a highly complex, three-dimensional structure that facilitates sediment accumulation and influences adjacent sediment environments through altered hydrodynamic regimes. Infaunal communities adjacent to different coral types, including reef-building scleractinian corals and individual colonies of octocorals, are known to exhibit higher macrofaunal densities and distinct community structure when compared to non-coral soft-sediment communities. However, the coral types have different morphologies, which may modify the adjacent sediment communities in discrete ways. Here we address: (1) how infaunal communities and their associated sediment geochemistry compare among deep-sea coral types (*Lophelia pertusa, Madrepora oculata,* and octocorals) and (2) do infaunal communities adjacent to coral habitats exhibit typical regional and depth-related patterns observed in the Gulf of Mexico (GOM). Sediment push cores were collected to assess diversity, composition, numerical abundance, and functional traits of macrofauna (>300 µm) across 450 kilometers in the GOM at depths ranging from 263–1,095 m. Macrofaunal density was highest in *L. pertusa* habitats, but similar between *M. oculata* and octocorals habitats. Density overall exhibited a unimodal relationship with depth, with maximum densities between 600 and 800 m. Diversity and evenness were highest in octocoral habitats; however, there was no relationship between diversity and depth. Infaunal assemblages and functional traits differed among coral habitats, with *L. pertusa* habitats the most distinct from both *M. oculata* and octocorals. These patterns could relate to differences in sediment geochemistry as *L. pertusa* habitats contained high organic carbon content but low proportions of mud compared to both *M. oculata* and octocoral habitats. Distance-based linear modeling revealed depth, mud content, and organic carbon as the primary factors in driving coral infaunal community structure, while geographic location (longitude) was the primary factor in functional trait composition, highlighting both the location and ecological differences of *L. pertusa* habitats from other coral habitats. Enhanced habitat structural complexity associated with *L. pertusa* and differences in localized hydrodynamic flow may contribute to the dissimilarities in the communities found among the coral types. Our results suggest a decoupling for infaunal coral communities from the typical depth-related density and diversity patterns present throughout soft-sediment habitats in the GOM, highlighting the importance of deep-sea corals in structuring unique communities in the nearby benthos.

## Introduction

Deep-sea corals create a complex three-dimensional structure that enhances local biodiversity, supporting diverse and abundant fish and invertebrate communities (Mortensen et al., 1995; Costello et al., 2005; Henry & Roberts, 2007; Ross & Quattrini, 2007; Buhl-Mortensen et al., 2010). In recent years, knowledge of the sphere of influence of deep-sea corals has expanded, with evidence that coral habitats also influence surrounding sediments (Mienis et al., 2012; Demopoulos, Bourque & Frometa, 2014; Fisher et al., 2014; Demopoulos et al., 2016). Deep-sea corals are capable of altering their associated biotic and abiotic environment, thus serving as ecosystem engineers (e.g., Jones, Lawton & Shachak, 1994). The depositional environment and associated hydrodynamic regime around coral habitats differ from the extensive expanses of soft-sediments that dominate the sea floor (e.g., Mienis et al., 2009a; Mienis et al., 2009b; Mienis et al., 2012), with the three-dimensional structure of the coral causing turbulent flows that enhance sediment accumulation adjacent to coral structures. The different hydrodynamics around corals likely affects the sediment geochemistry and in turn infaunal community structure and function (Demopoulos, Bourque & Frometa, 2014).

**Figure 1:**
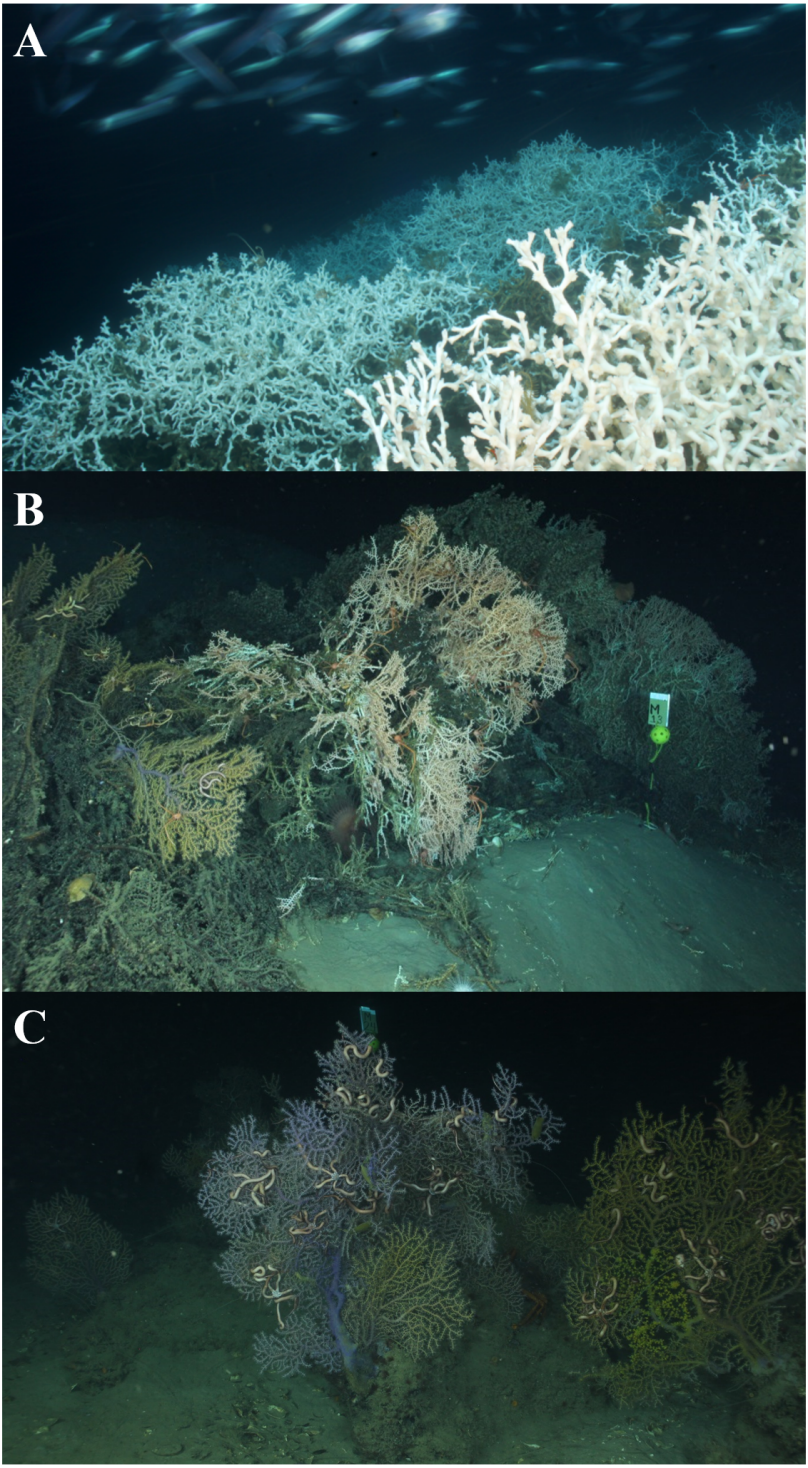
Coral habitat types. (A) *Lophelia pertusa* reef at VK906, Image credit: USGS/DISCOVRE. (B) *Madrepora oculata* colonies at AT357, Image credit: C. Fisher. (C) *Paramuricea* (Octocoral) colonies at MC203, Image credit: C. Fisher.

The northern Gulf of Mexico (GOM) contains a variety of deep-sea corals, including scleractinians, octocorals, and black corals, that represent a range of habitats. The most conspicuous is the scleractinian *Lophelia pertusa* (Fig. 1A) that occurs in a variety of forms, ranging from small colonies (∼1 to 2 m long and 1 to 2 m high; Brooke & Schroeder, 2007; Lunden, Georgian & Cordes, 2013) to large reefs (up to 600 m in length; Cordes et al., 2008; Lunden, Georgian & Cordes, 2013). It is the only deep-sea scleractinian that builds complex and continuous reef structures in the GOM, often containing co-occurring coral species, including *Madrepora oculata*, octocorals, and black corals. The deep-sea scleractinian, *M. oculata* (Fig. 1B)*,* is known to build reef structures in the northeast Atlantic (Davies et al., 2017); however, in the GOM it exists primarily as small or moderate-sized colonies. In general, the structure of *M. oculata* is more fragile than *L. pertusa* and fragments easily. Octocorals, including the genera *Paramuricea, Callogorgia,* and *Chrysogorgia*, occur as fan-like colonies attached to available hard substrate, and multiple colonies can occur in a small area (Fig. 1C). In the northern GOM, deep-sea corals generally occur on mounds of authigenic carbonate (Schroeder, 2002). As three-dimensional heterogeneous habitats, the complexity of the coral habitats have the potential to affect hydrodynamic regimes, and thus sediment accumulation, in different ways. Elevation above the benthic boundary layer into higher velocity laminar flows allows for increased availability of food resources (Buhl-Mortensen & Mortensen, 2005; Carney et al., 2005). Although hydrodynamic regimes and particle deposition have been investigated around *L. pertusa* reefs (Mienis et al., 2007; Mienis et al., 2009a; Mienis et al., 2009b; Mienis et al., 2012), little is known about the specific hydrodynamic regimes around *M. oculata* or octocoral habitats. Faunal associates are known to differ between different species of coral (Buhl-Mortensen & Mortensen, 2005); however, whether these differences extend to adjacent sediment communities is unknown.

Previous research on deep-sea coral-associated infaunal communities has focused on *L. pertusa* (Henry & Roberts, 2007; Demopoulos, Bourque & Frometa, 2014) and octocorals with respect to anthropogenic disturbance (e.g., oil spills, Fisher et al., 2014; Demopoulos et al., 2016). Demopoulos, Bourque & Frometa (2014) found that infaunal communities adjacent to *L. pertusa* habitat were distinctly different from nearby (>100 m) background soft-sediment habitats, with the amount of difference and the overall sphere of influence of a reef varying with size. Fisher et al. (2014) also demonstrated that infaunal communities adjacent to individual colonies and multiple mixed species of octocorals also differ from infauna in northern GOM non-coral soft sediments (Rowe & Kennicutt II, 2009; Wei et al., 2010). There is also evidence that infaunal communities are distinct between sites for both *L. pertusa* (Demopoulos, Bourque & Frometa, 2014) and octocorals (Demopoulos et al., 2016). However, both studies were based on a limited number of sites, so it is unclear whether these patterns can be generalized to the GOM region as a whole. These differences suggest additional large-scale factors (e.g., geographic separation and/or depth) and/or local-scale processes (e.g., near-bottom currents affecting grain size, food availability, and larval dispersal) are driving infaunal community patterns (Wei et al., 2010).

Infaunal soft-sediment communities in the northern GOM differ by geographic location and depth (Rowe & Kennicutt II, 2009; Wei et al., 2010). Density decreases with depth while taxa diversity exhibits a mid-depth (1,100–1,300 m) maximum (Rowe & Kennicutt II, 2009). Community composition is influenced by both geographic location and depth, with zones (as defined by Wei et al., 2010) encompassing specific depth ranges, ranging from 635 to 3,314 m, and separated into east and west components. These zones were correlated to detrital particulate organic carbon export flux, primarily from the Mississippi River (Wei et al., 2010), where particulate organic carbon (POC) flux decreases with depth (Biggs, Hu & Muller-Karger, 2008). The flux of POC has also been found to be higher in the northeast GOM than the northwest (Biggs, Hu & Muller-Karger, 2008), and consequently, biomass of infaunal communities is positively correlated with sediment organic carbon content (Morse & Beazley, 2008). However, given the complex hydrodynamic environments around deep-sea corals may substantially alter sediment organic matter deposition (Mienis et al., 2009a; Mienis et al., 2009b; Mienis et al., 2012), the typical depth/diversity relationship may be decoupled as a result of patchiness in sediment organic carbon content around deep-sea corals.

Despite the number of studies that have investigated patterns in deep-sea biodiversity (see reviews by Danovaro et al., 2008; Thurber et al., 2014), we are just beginning to understand local and regional patterns in biodiversity, community structure, and its associated controls. This study presents new data, in combination with published information, that directly addresses the role of habitat heterogeneity in structuring deep-sea diversity and whether generalizations in density, diversity, and community structure can be made regarding deep-sea coral proximal sediments. The primary objective of the study was to compare soft-sediment benthos adjacent to *L. pertusa*, *M. oculata*, and octocorals on a regional scale of the northern GOM. We tested three hypotheses: (1) macrofaunal abundance, taxa diversity, and composition of benthic communities adjacent to different coral types differ from one another; (2) macrofaunal abundance, taxa diversity, and composition of benthic communities adjacent to different coral types differ from nearby non-coral soft sediments; and (3) near-coral communities across all sites exhibit similar geographic and depth-related patterns to those of soft-sediment habitats in the northern GOM. These results have important implications for management of deep-sea coral environments, helping to better refine the sphere of influence for different coral types to determine whether a one-size-fits-all or a coral-specific approach is most appropriate for management strategies.

## Materials & Methods

### Study location

**Figure 2:**
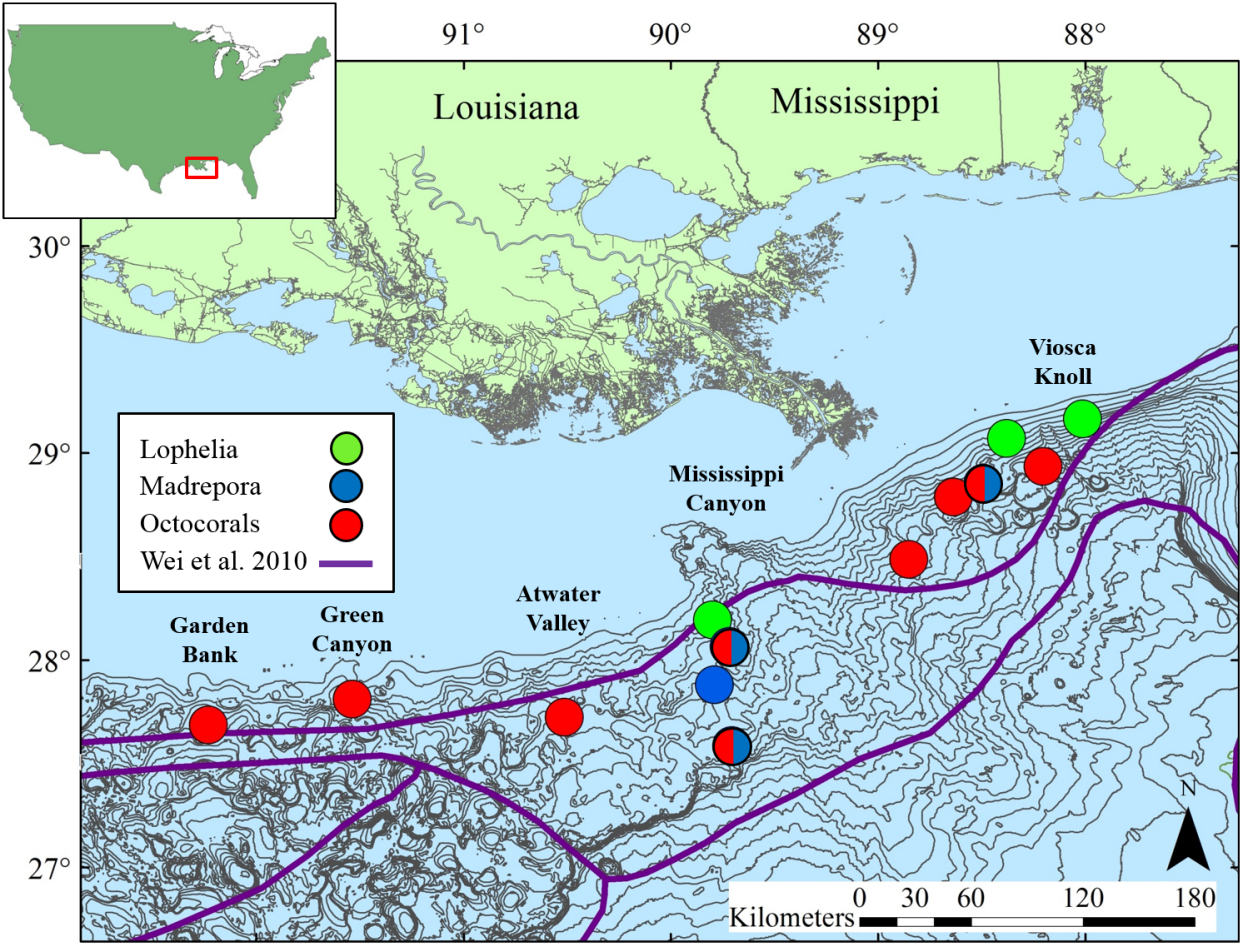
Map of study locations in the northern Gulf of Mexico, with soft-sediment deep-sea infaunal community zonation from Wei et al. (2010). Bathymetric intervals are 50 m starting at 150 m depth. Base map data from NOAA, GCOOS.

Sediments were collected adjacent to deep-sea corals at 13 sites in the northern GOM (Table  1, Fig. 2) at depths ranging 263 to 1,095 m. Site names correspond to Bureau of Ocean Energy Management (BOEM) Lease Block designations. Three types of coral habitats were sampled based on the dominant coral type at a given location: *L. pertusa* (*Lophelia*), *M. oculata* (*Madrepora*), and octocoral (Octocoral), where the primary component was either a single or mixed species of *Paramuricea*, *Callogorgia*, and/or *Chrysogorgia*.

**Table 1:** Locations, depths, gear used, number of cores used for infauna and sediment geochemistry analyses, and data source.

Coral	Site	Year	Vessel/ Vehicle	Latitude	Longitude	Depth (m)	Proximity	Infauna	Geo	Source
***Lophelia pertusa***	MC751	2009	RB/Jason	28.1937	−89.7988	440	Near	3		Demopoulos, Bourque & Frometa (2014)
	MC751	2009	RB/Jason	28.1987	−89.8010	431	Background	2		Demopoulos, Bourque & Frometa (2014)
	MC751	2009	SJ/BC	28.1969	−89.7988	429	Background	1		Demopoulos, Bourque & Frometa (2014)
	MC751	2009	SJ/BC	28.1968	−89.7999	427	Background	1		Demopoulos, Bourque & Frometa (2014)
	MC751	2010	RB/Jason	28.1940	−89.7983	439	Near		OC	This study
	VK906	2009	RB/Jason	29.0696	−88.3771	388	Near	3		Demopoulos, Bourque & Frometa (2014)
	VK906	2009	RB/Jason	29.0692	−88.3776	393	Near	3		Demopoulos, Bourque & Frometa (2014)
	VK906	2009	RB/Jason	29.0690	−88.3771	392	Near	3		Demopoulos, Bourque & Frometa (2014)
	VK906	2009	RB/Jason	29.0673	−88.3802	432	Background	3		Demopoulos, Bourque & Frometa (2014)
	VK906	2009	SJ/JSL	29.0691	−88.3761	393	Background	1		Demopoulos, Bourque & Frometa (2014)
	VK906	2009	SJ/BC	29.0727	−88.3781	418	Background	1		Demopoulos, Bourque & Frometa (2014)
	VK906	2010	RB/Jason	29.0697	−88.3770	390	Near		OC	This study
	VK906	2010	RB/Jason	29.0693	−88.3775	395	Near		OC	This study
	VK906	2011	HC	29.0699	−88.3776	402	Near	3	OC/GS	This study
	VK906	2011	HC	29.0694	−88.3775	392	Near	3	OC/GS	This study
	VK906/862	2010	RB/Jason	29.1066	−88.3842	314	Near		OC	This study
	VK906/862	2010	RB/Jason	29.0691	−88.3769	393	Near		OC	This study
	VK826	2009	RB/Jason	29.1578	−88.0162	475	Near	3		Demopoulos, Bourque & Frometa (2014)
	VK826	2009	RB/Jason	29.1582	−88.0168	470	Near	2		Demopoulos, Bourque & Frometa (2014)
	VK826	2009	RB/Jason	29.1587	−88.0104	480	Near	2		Demopoulos, Bourque & Frometa (2014)
	VK826	2009	SJ/BC	29.1701	−88.0133	470	Background	1		Demopoulos, Bourque & Frometa (2014)
	VK826	2009	SJ/BC	29.1707	−88.0123	461	Background	1		Demopoulos, Bourque & Frometa (2014)
	VK826	2009	SJ/BC	29.1677	−88.0132	472	Background	1		Demopoulos, Bourque & Frometa (2014)
	VK826	2009	SJ/BC	29.1708	−88.0113	458	Background	1		Demopoulos, Bourque & Frometa (2014)
	VK826	2010	RB/Jason	29.1649	−88.0116	461	Near		OC	This study
	VK826	2010	RB/Jason	29.1588	−88.0105	477	Near		OC	This study
	VK826	2011	HC/Mill	29.1650	−88.0115	464	Near	4	OC/GS	This study
***Madrepora oculata***	AT47	2009	RB/Jason	27.8802	−89.7888	840	Near	3		This study
	AT47	2009	RB/Jason	27.8898	−89.7944	837	Background	3		This study
	AT357	2011	HC/Mill	27.5865	−89.7045	1051	Near	3	OC/GS	This study
	AT357	2013	NA/Herc	27.5867	−89.7046	1,049	Near	3	GS	This study
	AT357	2013	NA/Herc	27.5857	−89.7050	1,054	Near	3	GS	This study
	MC118	2010	RB/Jason	28.8527	−88.4925	883	Near	3		This study
	MC118	2010	RB/Jason	28.8527	−88.4927	882	Near	3	OC	This study
	MC118	2011	HC/Mill	28.8526	−88.4926	887	Near	3	OC/GS	This study
	MC118	2013	NA/Herc	28.8528	−88.4927	884	Near	3	GS	This study
	MC118	2013	NA/Herc	28.8527	−88.4925	883	Near	1	GS	This study
	MC885	2010	RB/Jason	28.0664	−89.7170	633	Near	3	OC/GS	This study
	MC885	2014	AT/Alvin	28.0661	−89.7172	634	Near	1	GS	This study
**Octocoral**	GB299	2009	RB/Jason	27.6863	−92.2308	359	Near	5		This study
	GB299	2009	RB/Jason	27.6862	−92.2309	355	Background	5		This study
	GB299	2010	RB/Jason	27.6866	−92.2310	362	Near	3		This study
	GB299	2010	RB/Jason	27.6865	−92.2307	361	Near	3		This study
	GB299	2010	RB/Jason	27.6846	−92.2209	340	Near	2	OC/GS	This study
	GC140	2010	RB/Jason	27.8108	−91.5371	263	Near	2	OC	This study
	GC249	2010	RB/Jason	27.7241	−90.5142	789	Near	3	OC/GS	This study
	MC118	2011	HC/Mill	28.8560	−88.4936	888	Near	3	OC/GS	Demopoulos et al. (2016)
	MC203	2011	HC/Mill	28.7873	−88.6347	919	Near	3	OC/GS	Demopoulos et al. (2016)
	MC036	2011	HC/Mill	28.9354	−88.2014	1094	Near	3	OC/GS	Demopoulos et al. (2016)
	MC036	2011	HC/Mill	28.9354	−88.2027	1,094	Near	3	OC/GS	Demopoulos et al. (2016)
	MC036	2014	NA/Herc	28.9354	−88.2027	1,094	Near	4	GS	This study
	MC507	2011	HC/Mill	28.4855	−88.8508	1,043	Near	3	OC/GS	Fisher et al. (2014)
	MC885	2014	AT/Alvin	28.0661	−89.7173	635	Near	1	GS	This study
	AT357	2011	HC/Mill	27.5866	−89.7041	1,048	Near	3	OC/GS	Fisher et al. (2014)
	AT357	2014	AT/Alvin	27.5864	−89.7044	1,050	Near	1	GS	This study
	AT357	2014	AT/Alvin	27.5861	−89.7044	1,054	Near	1	GS	This study

**Notes.**

RB NOAA Ship *Ronald H. Brown* SJ R/V *Seward Johnson* HC *Holiday Chouest* NA E/V *Nautilus* AT R/V *Atlantis* Jason ROV *Jason II* BC Box core JSL HOV *Johnson Sea Link II* Mill ROV *Millenium* Herc ROV *Hercules* Alvin HOV *Alvin* Infauna number of cores used for infaunal analyses OC organic carbon content analysis performed GS grain size analysis performed

### Data collection

Sediment samples were collected between 2009 and 2014 on seven cruises in the GOM (Table 1). Near-coral push cores (6.35 cm diameter) were collected adjacent to coral habitats (within 1 m) using a remotely operated vehicle (ROV) or a human-occupied vehicle (HOV). Additional background, non-coral soft-sediment cores were collected via ROV (6.35 cm diameter) at >1,200 m away from *M. oculata* habitat at AT47 and >14 m away from octocoral habitat at GB299. All sediment cores were sectioned vertically (either 0–1, 1–3, 3–5, 5–10 cm or 0–2, 2–5, 5–10 cm) after recovery. Eight of the total 122 cores only penetrated 7–9 cm. Data for 0–5 cm sediment fractions from *L. pertusa* sites in 2009 were published in Demopoulos, Bourque & Frometa (2014) and the octocoral sites in 2011 were published in Demopoulos et al. (2016) and Fisher et al. (2014) (see Table 1). Sediment core sections processed for infauna analysis were preserved whole in an 8–10% buffered formalin solution until they were returned to the laboratory where they were stained with rose bengal and washed through a 300-µm sieve to retain the macrofauna. Macrofauna were sorted with a dissecting microscope and identified to the lowest practical taxonomic level, including family level for polychaetes, peracarid crustaceans, and aplacophorans. Sediment sections from cores for geochemistry analysis were frozen whole at −20 °C until returned to the lab. Grain size analysis was performed using the Folk method (Folk, 1968). Some sediments from 2010 and 2011 were processed for grain size and organic carbon content by Alpha Analytical Labs, with resultant data downloaded from the NRDA DIVER website (available at https://dwhdiver.orr.noaa.gov/) on December 9, 2013. All new data presented herein is available in Bourque & Demopoulos (2018).

### Data analyses

Abundance of individuals and univariate measures of biodiversity among near-coral cores, among background cores, and between near-coral and background cores for the three coral types were analyzed using one-way analysis of variance (ANOVA) followed by post-hoc test Tukey’s HSD for multiple comparisons. As background cores for *M. oculata* and octocorals were only sampled at one site each (GB299 and AT47), additional comparisons were made between near-coral and background cores at those sites only. All data were tested for normality and heteroscedasticity using Shapiro–Wilk and Levene’s test (Zar, 1999) and log_e_-transformed when necessary. If transformation did not achieve normality or heteroscedasticity, a non-parametric Kruskal–Wallis test was used on univariate measures followed by a pairwise Wilcox test with a Holm correction for multiple comparisons. Depth relationships with abundance and diversity measures were tested using linear and polynomial regressions. A significance level of *p* < 0.05 was used in all tests. Univariate statistics were computed with the program R (R Development Core Team, 2016). Diversity was examined using the Shannon-Wiener diversity index (H′log_e_), Pielou’s evenness (J′), and rarefaction (ES[n]) analysis, based on untransformed abundance data using DIVERSE in PRIMER Statistical Software version 7 (Clarke & Gorley, 2015).

Community structure was assessed by examining the overall contribution of higher level taxa, including Polychaeta, Oligochaeta, Crustacea, Mollusca, and Other Taxa. Other Taxa included Halacaridae, Callipallenidae, Cnidaria (Anthozoa, Hydrozoa), Echinodermata (Holothuroidea, Ophiuroidea), Nemertea, Urochordata, Chaetognatha, Sipuncula, Echiura, and Turbellaria. Colonial taxa (i.e., Porifera, Bryozoa, Octocorallia) were not included in abundance, diversity, and community analyses but were included in overall taxa numbers. Multivariate analysis of community structure across cores for coral habitats was performed on square-root transformed abundance data using Bray-Curtis similarities in PRIMER version 7 (Clarke & Gorley, 2015). Differences in infaunal communities with respect to coral type were examined using one-way analysis of similarity (ANOSIM). Similarity of percentages (SIMPER) was used to identify the taxa responsible for discriminating between communities and to assess the variability within communities. Variability among coral communities was assessed using multivariate dispersion (MVDISP). A subset of the multivariate communities was assessed in conjunction with geographic location (latitude, longitude), bathymetric position (depth), sediment grain size (mud content), and sediment organic carbon content using distance-based linear modelling (DISTLM) using the PERMANOVA + add on package to PRIMER 7 (Anderson, Gorley & Clarke, 2008) for locations where all of these data were available. DISTLM performs nominal tests of each variable’s explanatory power on community structure and builds a multivariate statistical model of explanatory power of a suite of variables when considered together to determine the “best” model based on the Akaike information criterion for small sample sizes (AICc). Results of density, diversity, and multivariate community analyses were examined after excluding cores that penetrated less than 10 cm, and their exclusion did not alter the outcome of these analyses, so they are included here to increase sample size.

Functional groups of taxa were broken down into four traits (feeding method, feeding location, motility, and living habit) encompassing 15 modalities. A trait matrix was created using a ‘fuzzy coding’ procedure (Chevenet, Dolédec & Chessel, 1994) based on published trait information. The ‘fuzzy coding’ allows flexibility in assigning taxa with a mixture of trait characteristics and exhibiting traits over different degrees (Chevenet, Dolédec & Chessel, 1994), while also capturing potential intraspecific variations in trait expression (Castella & Speight, 1996; Charvet et al., 2000). A scoring range of 0–3 was used, with 0 signifying no affinity to a modality and 3 representing a high affinity to a modality, then normalized within each trait (Bolam & Eggleton, 2014). A station by trait matrix was created by multiplying taxa abundance by trait values and then summing across each core. This matrix was then imported into PRIMER, square-root transformed, and a Bray-Curtis similarity matrix was created. The functional trait-weighted community data were analyzed using nMDS, one-way ANOSIM between coral habitats, and DISTLM with geographic and environmental variables.

## Results

### Near-coral habitats

**Figure 3:**
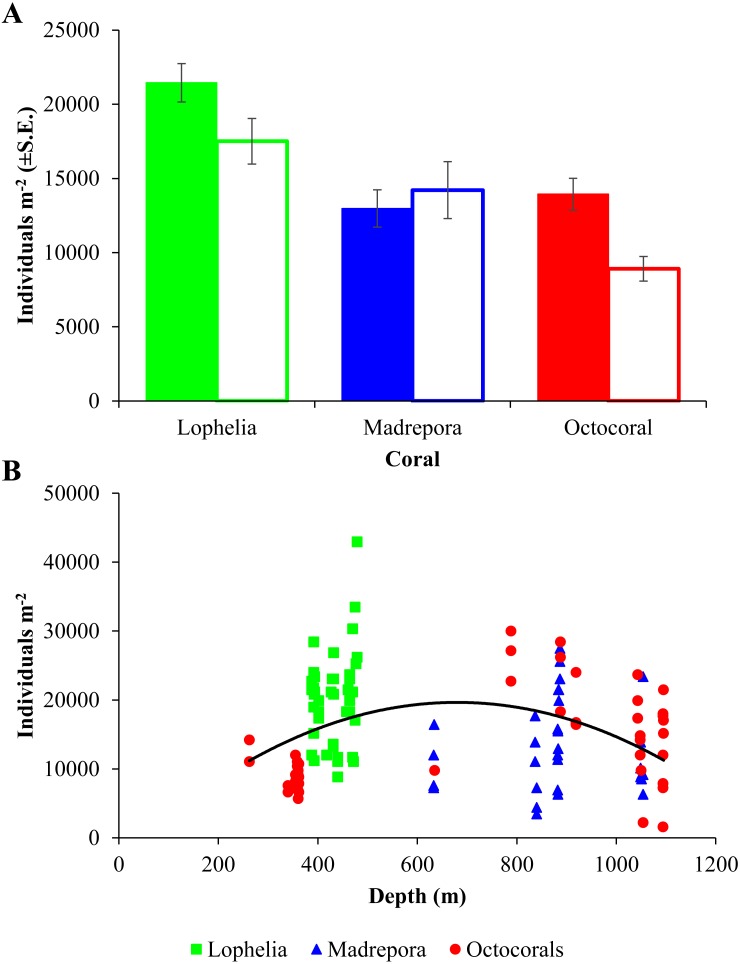
Macrofaunal density at deep-sea coral habitats. (A) Mean macrofaunal density (individuals m^−2^ ±  1 S.E.) near coral (solid bars) and background (open bars) soft-sediment habitats in the GOM. (B) Macrofaunal density (individuals m^−2^) of near-coral cores with depth, with polynomial trendline delineated (*y* =  − 0.0475*x*^2^ + 63.81*x* − 1579, *R*^2^ = 0.101).

Macrofaunal densities differed between the three coral habitats (One-way ANOVA, *F*_2,98_ = 13.52, *p* = 6.5e^−06^; Table 2; Fig. 3A) and ranged from 42,970 individuals m^−2^ at *L. pertusa* habitats (VK826) to 1,580 individuals m^−2^ near octocoral habitats (MC036). Mean macrofaunal density was significantly higher near *L. pertusa* habitats (21,452 ± 1,291 individuals m^−2^) than near either *M. oculata* (12,976 ± 1,256 individuals m^−2^) or octocoral habitats (13,939 ± 1,079 individuals m^−2^; Tukey HSD, *p* < 0.00005), while densities at *M. oculata* and octocoral habitats were similar (Tukey HSD, *p* = 0.83). Macrofaunal density exhibited a significant quadratic relationship with depth (Fig. 3B; *F*_2,98_ = 5.51, *p* = 0.005, *R*^2^ = 0.101), with a mid-depth maximum between 600 and 800 m and lower densities at both shallower and deeper depths.

**Table 2:** Community metrics for near-coral and background habitats. Macrofaunal density (individuals m^−2^), the total number of taxa, the estimated number of taxa (ES[n]), Shannon diversity (H′log_*e*_), taxa evenness (Pielou’s *J*′), and the multivariate dispersion (MVDISP). MVDISP was calculated among coral types and between near-coral and background samples within sites. Values in parentheses indicate 1 standard error.

Coral/site	*N*	Density (individuals m^−2^)		Total Taxa	ES (n)	H′log_*e*_		*J*′		MVDISP
**Lophelia**	**42**	**20,232**	**(1,040)**	**92**	**ES(920) 73.63**	**2.57**	**(0.05)**	**0.86**	**(0.01)**	**0.615**
Near-coral	29	21,452	(1,291)	84	ES(100) 32.56	2.58	(0.06)	0.86	(0.01)	0.628
MC751 Near-coral	3	10,532	(862)	33	ES(47) 23.31	2.69	(0.08)	0.94	(0.01)	1.6
MC751 Background	4	17,773	(2,539)	34	ES(47) 17.47	2.45	(0.04)	0.84	(0.03)	0.7
VK906 Near-coral	15	20,600	(1,019)	63	ES(47) 20.51	2.53	(0.09)	0.86	(0.01)	0.967
VK906 Background	5	18,797	(3,092)	55	ES(47) 23.41	2.72	(0.18)	0.88	(0.02)	1.352
VK826 Near-coral	11	25,592	(2,286)	64	ES(47) 20.03	2.62	(0.10)	0.83	(0.02)	0.91
VK826 Background	4	15,640	(2,549)	48	ES(47) 21.50	2.42	(0.07)	0.85	(0.01)	1.823
**Madrepora**	**32**	**13,092**	**(1,148)**	**74**	**ES(920) 66.58**	**2.39**	**(0.06)**	**0.88**	**(0.01)**	**1.201**
Near-coral	29	12,976	(1,256)	73	ES(100) 33.83	2.38	(0.07)	0.88	(0.02)	1.215
AT47 Near-coral	3	5,055	(1,139)	19	ES(47) 19.00	2.16	(0.13)	0.95	(0.01)	1.429
AT47 Background	3	14,218	(1,922)	24	ES(47) 16.05	2.46	(0.02)	0.91	(0.02)	0.571
**Octocoral**	**48**	**13,415**	**(994)**	**88**	**ES(920) 74.24**	**2.54**	**(0.05)**	**0.91**	**(0.01)**	**1.083**
Near-coral	43	13,939	(1,079)	86	ES(100) 37.35	2.54	(0.05)	0.90	(0.01)	1.097
GB299 Near-coral	13	8,458	(480)	49	ES(47) 21.72	2.43	(0.06)	0.92	(0.01)	1.028
GB299 Background	5	8,910	(832)	35	ES(47) 21.97	2.51	(0.09)	0.94	(0.01)	0.784

**Figure 4:**
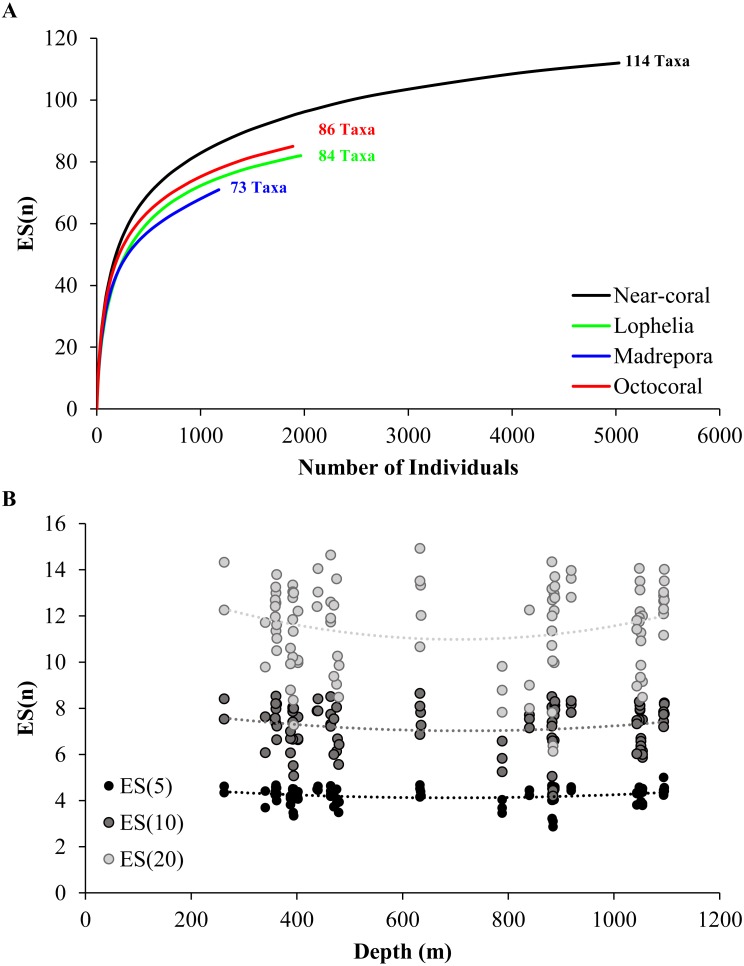
Rarefaction via estimated number of taxa (ES[n]). (A) Near-coral habitats pooled by coral type and all near-coral samples, with total number of taxa including colonial taxa indicated. (B) Rarefaction compared with depth for multiple levels of ES(n). ES(5): *y* = 2e^−06^*x*^2^ − 0.0021*x* + 4.8524, *R*^2^ = 0.041; ES(10): *y* = 3e^−06^*x*^2^ − 0.0044*x* + 8.5654, *R*^2^ = 0.025; ES(20): *y* = 8e^−06^*x*^2^ − 0.0109*x* + 14.726, *R*^2^ = 0.036.

A total of 114 taxa were observed near coral habitats across 5,029 individuals identified, with 86 taxa observed near octocorals, 84 taxa near *L. pertusa*, and 73 taxa near *M. oculata* habitats (Table 2). There was a large amount of taxa overlap between habitats, with 50 taxa (43.9%) shared among all three habitats, 29 taxa shared between any two habitats (25.4%), and 35 taxa (30.7%) present at only a single habitat. Diversity was highest near octocoral habitats for all diversity metrics assessed (Fig. 4A; Table 2). Although *M. oculata* habitats had overall lower Shannon diversity (Table 2), consistent with the rarefaction results (Fig. 4A), there was no significant difference among coral habitats (One-way ANOVA, *F*_2,98_ = 2.85, *p* = 0.063). However, there was a significant difference in evenness (*J*’, Kruskal test, *χ*^2^ = 9.87, *df* = 2, *p* = 0.007) among coral habitats with evenness higher in octocoral habitats than in *L. pertusa* habitats (*p* = 0.002). Diversity was highly variable at all depths (Fig. 4B), and there was no significant linear or quadratic relationship between diversity and depth for any of the metrics assessed (Shannon diversity, *p* > 0.46; evenness, *p* > 0.057; ES[n], *p* > 0.16).

**Figure 5:**
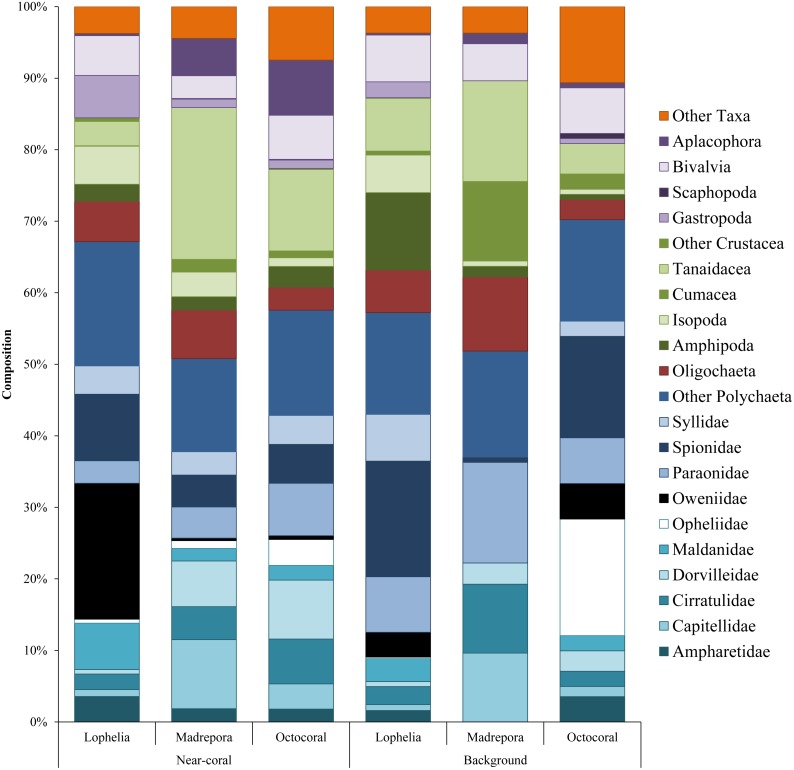
Taxonomic composition of dominant macrofauna at near-coral and background habitats. Other Taxa includes Halacaridae, Callipallenidae, Cnidaria, Echinodermata, Nemertea, Urochordata, Chaetognatha, Sipuncula, Echiura, and Turbellaria.

Overall infaunal composition varied among coral habitats (Fig. 5). Polychaetes dominated all habitats, but had the highest proportion in *L. pertusa* sediments (67.1%), with 57.6% in octocorals and 50.8% in *M. oculata* habitats. In contrast, *M. oculata* habitats had the highest proportion of crustaceans (28.3%) dominated by tanaids. *Lophelia pertusa* and octocoral habitats contained 11.7% and 16.6% crustaceans respectively. Octocoral habitats had the highest proportion of molluscs (15.1%), comprised of similar proportions of bivalves (6.2%) and aplacophorans (7.7%), followed by *L. pertusa* habitats (11.8%) and *M. oculata* habitats (9.7%). Octocorals also had the highest proportion of “Other Taxa” (7.5%), containing high proportions of Nemertea, Hydrozoa, and Echinodermata. *Lophelia pertusa* and *M. oculata* habitats had similar amounts of “Other Taxa” (3.8% and 4.5% respectively), with *L. pertusa* dominated by Sipuncula and Nemertea, while *M. oculata* was dominated by Nemertea, Halacaridae, and Sipuncula.

**Figure 6:**
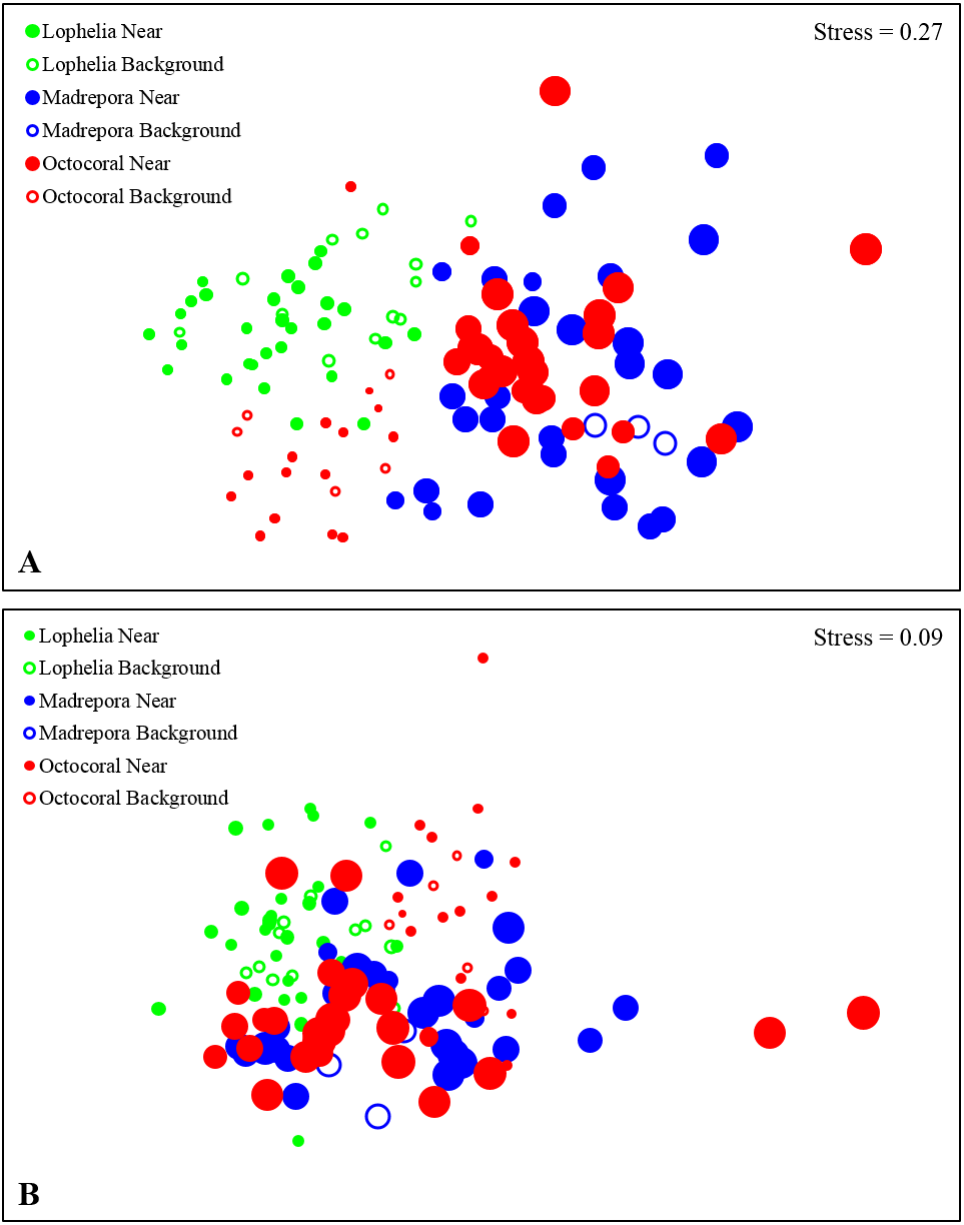
Non-metric multidimensional scaling (nMDS) of infaunal community composition and functional trait composition of near-coral and background habitats. (A) Infaunal community composition near coral habitats and in nearby background soft-sediments, based on Bray–Curtis similarities of square-root transformed abundance data from sediment core. (B) Functional trait composition of near-coral habitats and in nearby background soft-sediments, based on Bray–Curtis similarities of square-root transformed trait-weighted abundance data from sediment cores. Bubble size represents sample depth, ranging 263–1,095 m.

Macrofaunal community structure differed between all three coral habitats (Fig. 6A, One-way ANOSIM, *R* = 0.33, *p* = 0.0001). *Lophelia pertusa* communities were the most distinct group, with the largest difference from *M. oculata* (*R* = 0.56, *p* = 0.0001), followed by octocorals (*R* = 0.39, *p* = 0.0001). *Madrepora oculata* and octocoral habitats were also significantly different, although with a lower R value (*R* = 0.12, *p* = 0.002). Within group similarity was highest for *L. pertusa* (44.27%), followed by octocorals (34.19%) and *M. oculata* (31.77%). Multivariate dispersion followed a similar pattern, with the lowest dispersion within *L. pertusa* habitats and the highest dispersion within *M. oculata* habitats (Table 2). Six to seven taxa were responsible for greater than 50% of the similarity within coral habitats (Table 3), comprised primarily of polychaetes. Spionidae polychaetes (7.4–12.9%) and Bivalvia (5.4–10.7%) accounted for a high amount of similarity for all habitats. Dominance was also highest within *L. pertusa* habitats and lowest in octocoral habitats. Several of the taxa responsible for the most similarity within coral habitats represented community dominants (Table 3) and were responsible for the highest proportions of dissimilarity between habitats (Table 4). High densities of the polychaete family Oweniidae contributed most to the dissimilarity separating *L. pertusa* habitats from both *M. oculata* and octocoral habitats. Additionally, high densities of Maldanidae, Spionidae, and Gastropoda also distinguished *L. pertusa* habitats from *M. oculata* and octocoral habitats. Between *M. oculata* and octocoral habitats, higher densities of Pseudotanaidae, Capitellidae, and Tubificidae occurred in *M. oculata* habitats, while octocorals had higher densities of Dorvilleidae (Table 4). Within coral habitats, there was also a significant separation of communities by site (*L. pertusa*: ANOSIM, *R* = 0.36, *p* = 0.0003; *M. oculata*: ANOSIM, *R* = 0.54, *p* = 0.0001; Octocoral: ANOSIM, *R* = 0.50, *p* = 0.0001).

**Table 3:** Similarity percentage (SIMPER, %) results within coral habitats, including the taxa accounting for a cumulative 50% of the total similarity and total percent dominance (Dom). SIMPER analysis based on square-root transformed density data.

Near								
Lophelia	Avg. Similarity 44.27		Madrepora	Avg. Similarity 31.77		Octocorals	Avg. Similarity 34.19	
Taxa	%	Dom	Taxa	%	Dom	Taxa	%	Dom
Oweniidae	14.24	18.99	Paraonidae	12.05	4.37	Paraonidae	13.14	7.33
Spionidae	12.86	9.29	Tubificidae	8.15	6.80	Bivalvia	10.69	6.17
Bivalvia	8.09	5.54	Spionidae	7.38	4.45	Spionidae	8.58	5.43
Syllidae	6.8	3.96	Capitellidae	7.08	9.66	Cirratulidae	8.18	6.27
Maldanidae	6.6	6.50	Cirratulidae	6.8	4.62	Dorvilleidae	5.09	8.22
Tubificidae	6.52	5.64	Pseudotanaidae	6.32	13.85	Syllidae	4.93	4.06
			Bivalvia	5.38	3.19			

Background								
Lophelia	Avg. Similarity 40.79		Madrepora	Avg. Similarity 61.65		Octocorals	Avg. Similarity 47.15	
Taxa	%	Dom	Taxa	%	Dom	Taxa	%	Dom
Spionidae	18.1	16.15	Pseudotanaidae	15.47	14.07	Opheliidae	20.19	16.31
Syllidae	13.68	6.54	Paraonidae	14.38	14.07	Spionidae	19.88	14.18
Paraonidae	11.27	7.77	Capitellidae	12.2	9.63	Bivalvia	13.85	6.38
Bivalvia	6.88	6.54	Cirratulidae	10.88	9.63			
Colletteidae	5.3	2.72						

**Table 4:** SIMPER results of dissimilarity (%) between near-coral habitats with the taxa accounting for more than 3% of the dissimilarity and their associated mean densities (individuals m^−2^) for each habitat. SIMPER analysis based on square-root transformed density data.

**Lophelia/Madrepora**	** **	**Avg. Dissimilarity 74.17**	
**Taxa**	**%**	**Lophelia**	**Madrepora**
Oweniidae	7.51	4074.7	43.6
Spionidae	3.63	1993.8	577.4
Maldanidae	3.58	1394.6	228.8
Pseudotanaidae	3.55	65.4	1797.7
Gastropoda	3.52	1274.7	152.5
Tubificidae	3.29	1209.3	882.5
Capitellidae	3.12	207.0	1252.9
Bivalvia	3.04	1187.6	414.0
**Lophelia/Octocoral**	** **	**Avg. Dissimilarity 70.47**	
**Taxa**	**%**	**Lophelia**	**Octocoral**
Oweniidae	7.34	4074.7	73.5
Gastropoda	3.51	1274.7	161.7
Maldanidae	3.39	1394.6	286.6
Tubificidae	3.28	1209.3	448.2
Spionidae	3.27	1993.8	756.8
**Madrepora/Octocoral**		**Avg. Dissimilarity 69.37**	
**Taxa**	**%**	**Madrepora**	**Octocoral**
Pseudotanaidae	4.86	1797.7	551.1
Dorvilleidae	4.1	828.0	1146.3
Capitellidae	4.02	1252.9	492.3
Tubificidae	3.7	882.5	448.2
Cirratulidae	3.4	599.2	874.4
Bivalvia	3.23	414.0	859.7
Spionidae	3.14	577.4	756.8
Syllidae	3.03	424.9	565.8

**Figure 7:**
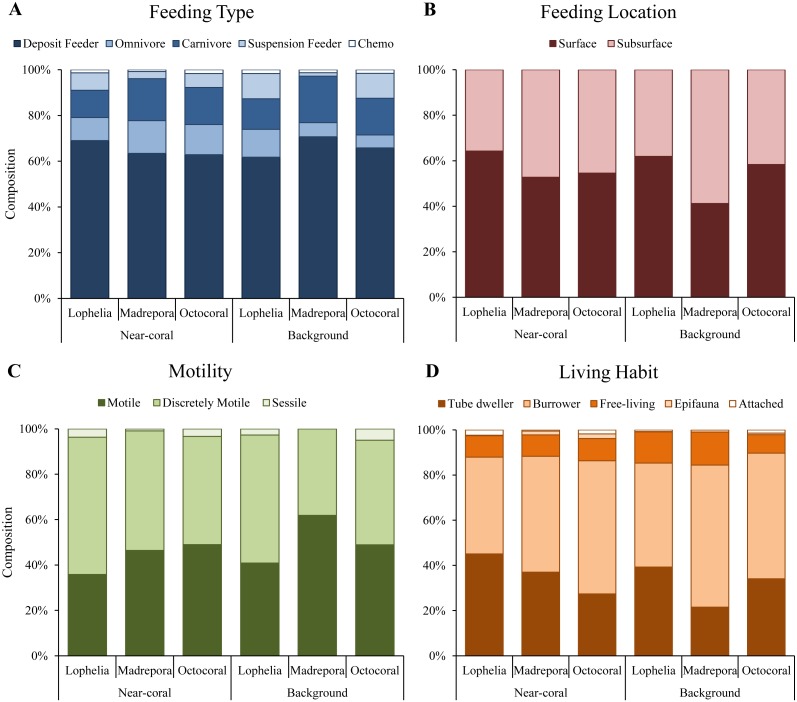
Functional trait composition of near-coral and background habitats. (A) Feeding type. (B) Feeding location. (C) Taxa motility. (D) Life habit.

Functional trait composition differed between coral habitats (Fig. 6B; One-way ANOSIM, *R* = 0.13, *p* = 0.0003), with *L. pertusa* habitats significantly different from both *M. oculata* (ANOSIM, *R* = 0.28, *p* = 0.0001) and octocoral habitats (ANOSIM, *R* = 0.14, *p* = 0.0008) while *M. oculata* and octocoral trait composition was similar (ANOSIM, *R* = 0.023, *p* = 0.2). SIMPER analysis indicated that *L. pertusa* habitats were distinct due to higher abundances of discretely motile, burrowing or tube-dwelling, surface deposit feeders (Fig. 7; Table 5). *Lophelia pertusa* habitats also contained higher proportions of attached and suspension-feeding taxa (Fig. 7), while *M. oculata* and octocoral habitats had higher proportions of motile and carnivorous taxa (Fig. 7).

**Table 5:** SIMPER results of dissimilarity (%) between functional traits in near-coral habitats comprising >50% of the cumulative the dissimilarity and trait-weighted mean densities (individuals m^−2^) at each habitat.

**Lophelia/madrepora**	** **	**Avg. Dissimilarity 21.89**	
**Taxa**	**%**	**Lophelia**	**Madrepora**
Surface	11.61	13,845	6,826
Tube dweller	11.5	9,717	4,806
Discretely motile	11.27	13,030	6,834
Deposit feeder	10.85	14,850	8,203
Burrower	6.82	9,221	6,656
**Lophelia/octocoral**	** **	**Avg. dissimilarity 21.52**	
**Taxa**	**%**	**Lophelia**	**Octocoral**
Discretely motile	12.05	13,030	6,650
Tube dweller	12.03	9,717	3,813
Surface	11.04	13,845	7,568
Deposit feeder	10.5	14,850	8,720
Burrower	6.71	9,221	8,222
**Madrepora/octocoral**	** **	**Avg. Dissimilarity 20.99**	
**Taxa**	**%**	**Madrepora**	**Octocoral**
Discretely motile	10.05	6,834	6,650
Surface	9.79	6,826	7,568
Tube dweller	9.66	4,806	3,813
Deposit feeder	8.98	8,203	8,720
Burrower	8.24	6,656	8,222
Subsurface	7.81	6,107	6,304

### Background habitats at coral sites

Macrofaunal densities in background sediments also differed among coral habitats (Fig. 3A; Table 2; One-way ANOVA, *F*_2,18_ = 8.77, *p* = 0.002), with background sediments at *L. pertusa* sites greater than those at the octocoral sites (Tukey HSD, *p* = 0.0015), while densities at the *M. oculata* sites were similar to both *L. pertusa* and octocorals (*p* > 0.1). Background sediments for *M. oculata* and octocoral habitats were collected at only one site each (AT47 and GB299). *Madrepora oculata* background sediments had higher densities than near-coral sediments (Table 2; AT-47; One-way ANOVA, *F*_1,4_ = 16.82, *p* = 0.015), while there was no difference between near-coral and background sediments at the octocoral site (Table 2; GB-299; One-way ANOVA, *F*_1,16_ = 0.24, *p* = 0.63).

There was high overlap in taxa between near-coral and background cores. At *M. oculata* site AT47, 10 taxa (30.3%) were shared between near-coral and background cores, while at octocoral site GB299, 30 taxa (55.6%) were shared between near-coral and background cores. Higher similarity between octocoral and background cores may be due to the close proximity of near-coral to background cores (>14 m). Shannon diversity did not differ among background sediments for the three coral habitats (One-way ANOVA, *F*_2,98_ = 0.15, *p* = 0.86). Within sites, there was no difference in Shannon diversity for *M. oculata* samples (Table 2; AT47; One-way ANOVA, *F*_1,4_ = 5.38, *p* = 0.081) or octocorals (Table 2; GB299; One-way ANOVA, *F*_1,16_ = 0.58, *p* = 0.46), with similar results when compared across all sampling locations (*M. oculata*; Kruskal test, *χ*^2^ = 0.30, *df* = 1, *p* = 0.58; Octocoral; One-way ANOVA, *F*_1,46_ = 0.044, *p* = 0.84).

Similar to near-coral habitats, background sediment communities differed between the three coral types (Fig. 6A; One-way ANOSIM, *R* = 0.64, *p* = 0.0001). However, unlike the near-coral communities, *M. oculata* background sediments were the most distinct from both octocoral (*R* = 0.98, *p* = 0.018) and *L. pertusa* (*R* = 0.77, *p* = 0.002) background communities. *Lophelia pertusa* and octocoral background communities were also distinct from one another (*R* = 0.50, *p* = 0.0006). *Madrepora oculata* background communities had the highest average similarity (SIMPER, 61.7%), followed by octocorals (47.2%) and *L. pertusa* (40.8%). The lower average similarity among *L. pertusa* background cores is likely a result of the group containing cores from three different *L. pertusa* sites. Similarity within background habitats was structured by fewer taxa than for near-coral habitats (Table 3). Dissimilarity of *M. oculata* background communities from the other coral habitats was structured by high densities of Pseudotanaidae, Capitellidae, Leuconidae, and Cirratulidae and low densities of Syllidae, Spionidae, and Opheliidae (Table 6).

**Table 6:** SIMPER results of dissimilarity (%) between background habitats with the taxa accounting for more than 3% of the dissimilarity and mean densities (individuals m^−2^) at each habitat. SIMPER analysis based on square-root transformed density data.

**Lophelia/Madrepora**	** **	**Avg. Dissimilarity 75.07**	
**Taxa**	**%**	**Lophelia**	**Madrepora**
Spionidae	6.15	2,828.1	105.3
Pseudotanaidae	5.55	145.8	2,001.1
Syllidae	4.72	1145.6	0.0
Capitellidae	4.19	145.8	1,369.1
Tubificidae	4.02	1,045.1	1,474.5
Leuconidae	3.98	0.0	1,053.2
Cirratulidae	3.51	437.5	1,369.1
Lumbrineridae	3.03	194.4	842.5
**Lophelia/Octocoral**	** **	**Avg. Dissimilarity 66.92**	
**Taxa**	**%**	**Lophelia**	**Octocoral**
Opheliidae	6.09	38.7	1,453.4
Syllidae	4.46	1,145.6	189.6
Spionidae	3.71	2,828.1	1,263.8
Tubificidae	3.34	1,045.1	252.8
Paraonidae	3.33	1,361.0	568.7
Desmosomatidae	3.08	559.0	63.2
Oweniidae	3.06	606.5	442.3
**Madrepora/Octocoral**		**Avg. Dissimilarity 73.99**	
**Taxa**	**%**	**Madrepora**	**Octocoral**
Pseudotanaidae	7.2	2,001.1	63.2
Opheliidae	6.54	0.0	1,453.4
Capitellidae	5.12	1,369.1	126.4
Spionidae	5.04	105.3	1,263.8
Leuconidae	4.9	1,053.2	0.0
Cirratulidae	4.87	1,369.1	189.6
Tubificidae	4.35	1,474.5	252.8
Lumbrineridae	4.15	842.5	126.4
Paraonidae	4.13	2,001.1	568.7
Dorvilleidae	3.37	421.3	252.8

Although within a site, *M. oculata* near-coral and background communities appear to be different due to the high R value (One-way ANOSIM, *R* = 0.85, *p* = 0.1), additional samples at AT47 would be needed to achieve statistical significance less than 0.1. Near-coral sediments at AT47 had very low proportions of polychaetes (16.6%), but high proportions of isopods (12.5%), tanaids (20.8%), aplacophorans (10.4%), and “Other Taxa” (14.6%), while polychaetes were proportionally dominant (51.9%) in background sediments. For octocoral communities there was no significant difference between near-coral and background communities at GB299 (One-way ANOSIM, *R* =  − 0.20, *p* = 0.94), likely influenced by the short distance (>14 m) between background and near-coral habitats. Near-coral and background sediments at GB299 had similar proportions of polychaetes (66.1 and 70.2% respectively) and molluscs (8.0 and 8.5% respectively), while near-coral sediment had higher proportions of “Other Taxa” (17.5%) than background sediments (10.6%).

Functional traits also differed among background samples of the three coral types (Fig. 6B; One-way ANOSIM, *R* = 0.47, *p* = 0.0001), with octocoral communities the most distinct from both *L. pertusa* (One-way ANOSIM, *R* = 0.51, *p* = 0.0009) and *M. oculata* habitats (One-way ANOSIM, *R* = 0.79, *p* = 0.018). Octocorals were distinguished from *M. oculata* habitats by lower densities of motile burrowers and subsurface deposit-feeders (Fig. 7; 43.9% dissimilarity) and from *L. pertusa* habitats by lower densities of discretely motile tube-dwellers and surface deposit feeders (Fig. 7; 41.0% dissimilarity). Functional trait composition also differed between *M. oculata* and *L. pertusa* habitats (One-way ANOSIM, *R* = 0.34, *p* = 0.013) with *M. oculata* habitat containing lower densities of discretely motile tube-dwellers, surface, and suspension-feeders (Fig. 7; 42.0% dissimilarity) than *L. pertusa* habitats. Similar to the results of the infaunal community analysis, near-coral and background functional trait composition of *M. oculata* habitats at AT47 are likely different (One-way ANOSIM, *R* = 0.89, *p* = 0.1), while those at octocoral site GB299 did not differ (One-way ANOSIM, *R* =  − 0.14, *p* = 0.85). At individual *L. pertusa* sites, there was no difference in functional trait composition between near-coral and background sediments (One-way ANOSIM, *R* < 0.52, *p* > 0.064).

### Environmental parameters contributing to macrofaunal community patterns

**Figure 8:**
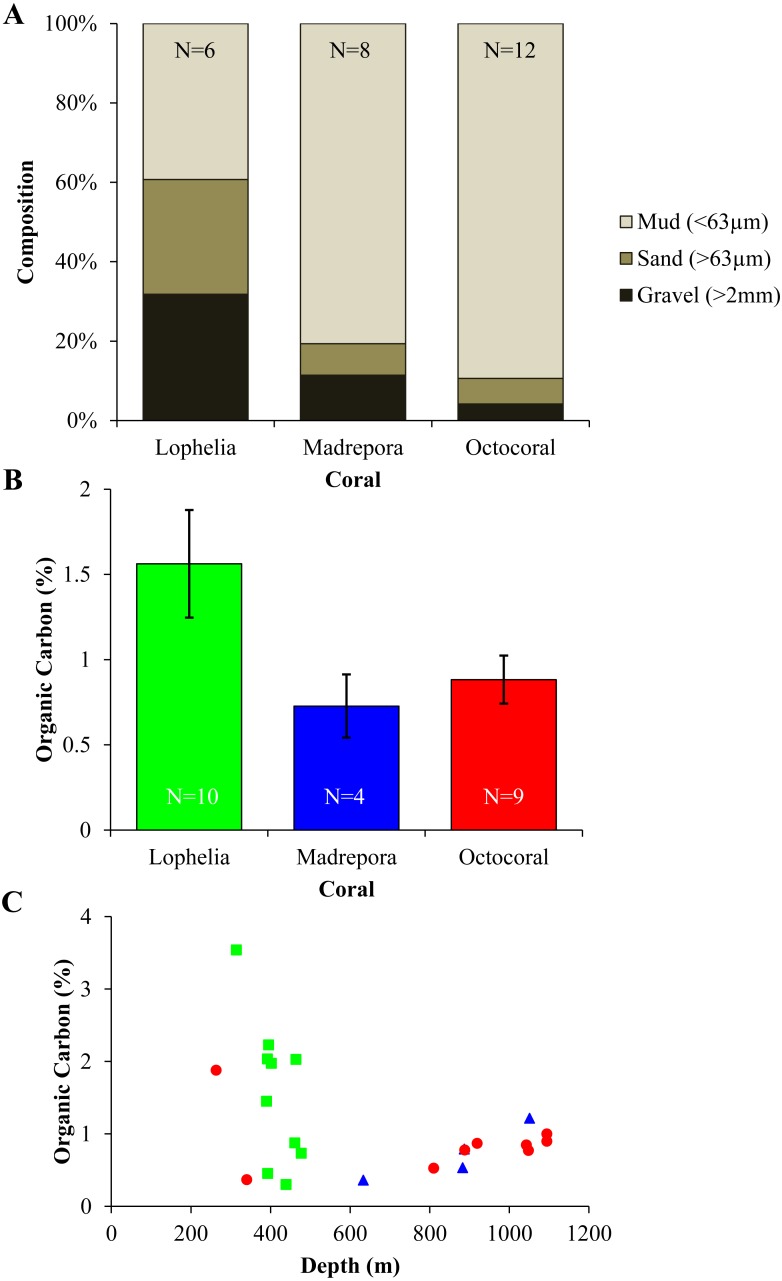
Sediment geochemistry for near-coral habitats. (A) Grain size composition. (B) Mean organic carbon content (% ± 1 S.E.). (C) Organic carbon content (%) with depth.

Sediment grain size and organic carbon content differed between the three coral habitats (Fig. 8). Grain size differed among coral habitats (Fig. 8A; Mud: One-way ANOVA, *F*_2,23_ = 27.36, *p* < 0.0001) with *L. pertusa* having significantly less mud (39.6%) than either *M. oculata* (80.7%) or octocoral (89.6%) sediments (Tukey HSD, *p* < 0.0001), while *M. oculata* and octocoral sediments had similar proportions of mud (Tukey HSD, *p* = 0.34). In contrast, *L. pertusa* sediments had higher proportions of gravel (>2 mm) grain size (32.0%) than either *M. oculata* (11.4%) or octocoral (4.2%) sediments. Although mean organic carbon content of sediments was higher in *L. pertusa* habitats than in both *M. oculata* and octocoral habitats (Fig. 8B), there was no significant difference among the coral habitats (Kruskal test, *χ*^2^ = 3.06, *p* = 0.22). The range of organic carbon content was highest for *L. pertusa* sediments (0.30–3.54%), followed by octocorals (0.37–1.88%) and *M. oculata* sediments (0.36–1.22%). However, there was no significant correlation of organic carbon content with depth (Fig. 8C; Spearman correlation, *ρ* =  − 0.22, *p* = 0.32).

**Figure 9:**
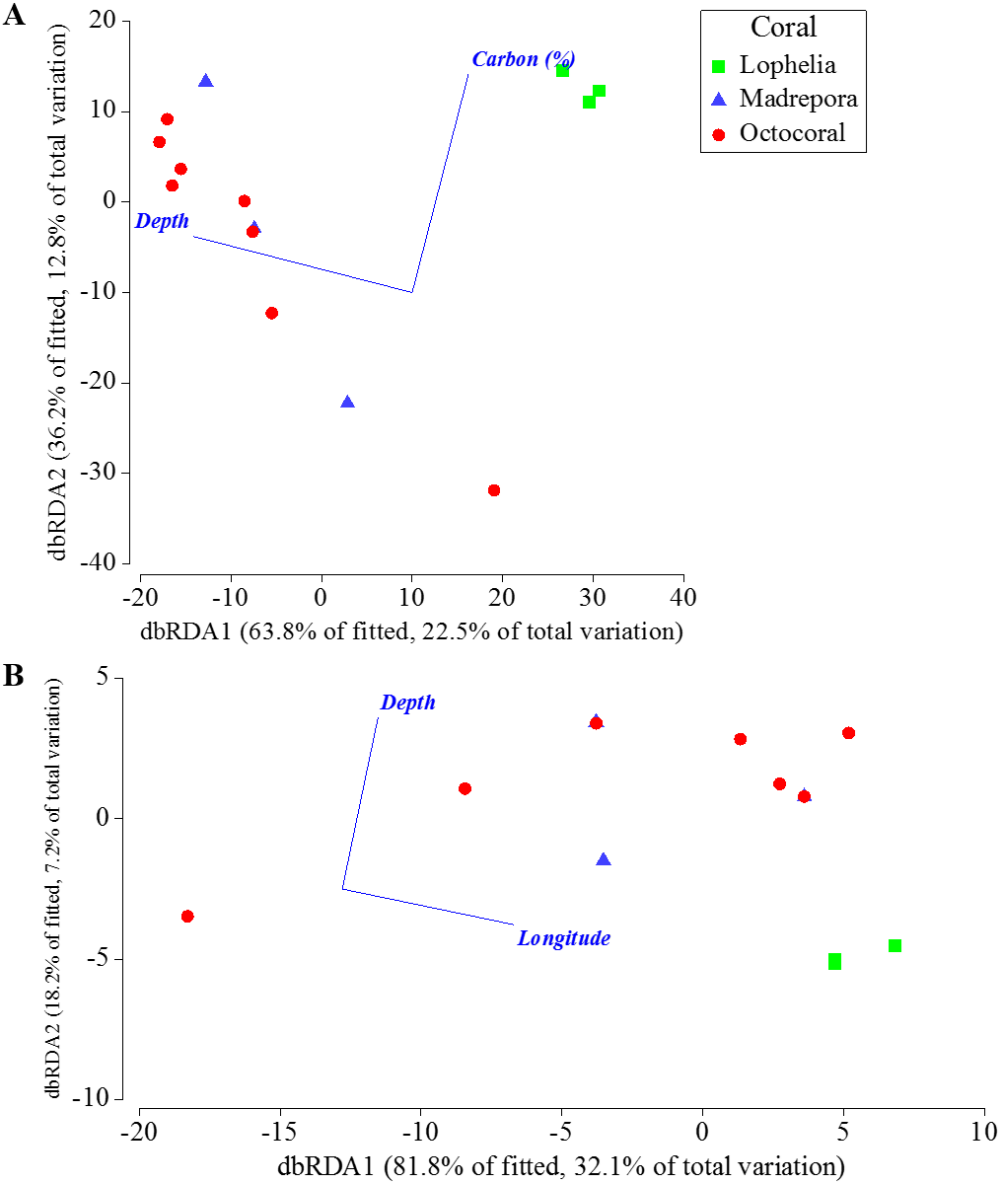
Distance-based redundancy analysis of the best two-variable model from distance-based linear modeling of sampling locations near coral habitats where sediment geochemistry data were available. (A) Based on Bray–Curtis similarities of square-root transformed abundance data averaged for individual sampling locations. (B) Based on Bray–Curtis similarities of square-root transformed functional trait weighted abundances averaged for individual sampling locations.

Depth, percent mud content, and percent organic carbon all individually explained a significant portion (DISTLM: 16.5–21.9%, *p* < 0.0061) of the macrofaunal community variation among near-coral cores (Table 7). The “best” model was depth alone, as suggested by the separation of communities by depth in the nMDS (Fig. 6A), followed by percent mud content (Table 7). The “best” two variable model included depth and percent organic carbon, explaining 35.2% of the community variation (Fig. 9A) and was within 1 unit of the best AICc value suggesting an equally probable model. DISTLM of functional trait composition with geographic and environmental variables differed from those of the macrofaunal community. Latitude and longitude were the only variables that individually explained a significant portion of the variation (Table 8; DISTLM: 22.3–32.1%, *p* < 0.047). The “best” model included longitude alone (Table 8), followed by the “best” two-variable model that included depth and longitude (Fig. 9B), which could explain a combined 39.2% of the variation in functional trait composition.

**Table 7:** Results from the distance-based linear modeling (DISTLM) of geographic, bathymetric, and environmental variables for near-coral communities using the AICc criteria and “best” model selection. Values in bold indicate variable explains a significant portion of the community variation.

Variable	SS(trace)	Pseudo-F	*p*	Proportion
Depth	4271.3	3.3641	**0.0001**	0.2190
Latitude	2361.4	1.6527	0.0538	0.1211
Longitude	2333	1.6303	0.0677	0.1196
% Mud	3600.7	2.7163	**0.0027**	0.1846
% Carbon	3228.5	2.3799	**0.0068**	0.1655
Total	19,507			

AICc	*R*^2^	RSS	No. Vars	Selections
102.98	0.21896	15,236	1	Depth
103.59	0.18458	15,907	1	% Mud
103.67	0.35256	12,630	2	Depth, % Carbon
103.91	0.1655	16,279	1	% Carbon
103.94	0.33989	12,877	2	Depth, % Mud
103.96	0.33903	12,894	2	Depth, Longitude
104.09	0.3329	13,013	2	Depth, Latitude
104.46	0.31499	13,363	2	Longitude, % Mud
104.5	0.31275	13,406	2	Longitude, % Carbon
104.64	0.12105	17,146	1	Latitude

**Table 8:** Results from the distance-based linear modeling (DISTLM) of geographic, bathymetric, and environmental variables for functional traits of near-coral communities using the AICc criteria and “best” model selection. Values in bold indicate variable explains a significant portion of the community variation.

Variable	SS(trace)	Pseudo-*F*	*p*	Proportion
Depth	160.25	1.0725	0.3322	0.0820
Latitude	436.03	3.4486	**0.0447**	0.2232
Longitude	626	5.6589	**0.0163**	0.3205
% Mud	165.87	1.1136	0.3077	0.0849
% Carbon	245.26	1.7232	0.1845	0.1256
Total	1953.3			

AICc	*R*^2^	RSS	No. Vars	Selections
68.817	0.32046	1327.3	1	Longitude
70.562	0.39229	1187	2	Depth, Longitude
70.676	0.38733	1196.7	2	Longitude, % Mud
70.689	0.22323	1517.2	1	Latitude
70.815	0.38119	1208.7	2	Longitude, % Carbon
71.332	0.35794	1254.1	2	Latitude, Longitude
71.936	0.32961	1309.4	2	Depth, Latitude
72.347	0.12557	1708	1	% Carbon
72.971	0.2782	1409.9	2	Latitude, % Mud
72.983	0.084919	1787.4	1	% Mud

## Discussion

*Lophelia pertusa*, *M. oculata*, and octocoral infaunal communities were distinctly different from one another, with *L. pertusa* habitats the most distinct from the other two, particularly for density and community structure. Two of the primary taxa separating *L. pertusa* communities from the other coral types were high abundance of the tube-building polychaete families Oweniidae and Maldanidae. While maldanids have tubes consisting of a membranous lining covered with mud, sand, or shells, oweniids build tubes of uniform sand-sized sediments. The increased composition of sand and gravel sediments at *L.  pertusa* habitats provides appropriate tube-building material, and suggests higher current velocity environments than at the other two coral habitats. The distinctness of *L. pertusa* habitats was also apparent in the functional trait analysis, with higher proportions of attached, tube-dwellers, and suspension feeders indicative of availability of hard substrata and high currents. Higher organic carbon content and lower proportions of mud have been shown to influence infaunal community composition (Gage & Tyler, 1991), which were sediment characteristics found in *L. pertusa* habitats. *Lophelia pertusa* habitats also exhibited the highest taxa dominance, indicative of a more stressful local environment. However, the low proportion of opportunistic taxa (e.g., Capitellidae, Cirratulidae) in sediments with high organic carbon content suggests these habitats do not experience pulsed organic enrichment (although peaks are known to occur; Mienis et al., 2012), but an overall high food availability. The high organic carbon content observed near *L. pertusa* is consistent with previous work in GOM *L. pertusa* habitats (Mienis et al., 2012). High proportions of mud, as observed at *M. oculata* and octocoral habitats, are known to inhibit dissolved oxygen concentrations (cf. Aller, 1982) and thus affect the suitability of deeper sediments. High proportions of Capitellidae, Cirratulidae, and Dorvilleidae, all tolerant of reducing environments, were present at *M. oculata* and octocoral habitats, consistent with lower pore water oxygenation at these habitats. The similar sediment characteristics (e.g., mud and organic content) found at *M. oculata* and octocoral habitats help explain that there were few differences observed in the infaunal communities.

The depth distribution of the three coral types may have influenced the observed community and functional differences between corals, especially for *L. pertusa. Lophelia pertusa* habitats occupied the narrowest depth range, which did not overlap with samples from the other two coral types and may limit our ability to separate depth from habitat differences. Shallower samples were collected for octocoral habitats, but not within the range of the *L. pertusa* habitats. *Lophelia pertusa* habitats are known to occur between 300 and 600 m in the GOM (Schroeder, 2002; Georgian, Shedd & Cordes, 2014), while deep-water octocorals are known to occur from 200 to 3,000 m (Cairns & Bayer, 2009; Quattrini et al., 2014). *Madrepora oculata* can co-occur with both *L. pertusa* and octocorals, with observed depths of 300–1,400 m (Schroeder et al., 2005), and the depth range for *M. oculata* core samples overlapped with octocoral samples. While macrofaunal densities in *M. oculata* and octocoral cores were similar despite their larger depth range, they were lower than those found at the shallower *L. pertusa* habitats. The distinct difference in community structure of *L. pertusa* sediments from other coral communities combined with their narrow depth range potentially heavily influenced the importance of depth in the DISTLM analysis. Additional samples of all three coral habitats that encompass their full depth range would help differentiate the roles that depth versus coral habitat may play in structuring these communities.

Another key factor that distinguished the three types of coral habitats is likely the habitat heterogeneity (i.e., patch size) of individual coral habitats and its effect on the local hydrodynamic regime. Varying patch sizes are known to influence sediment community structure in coastal settings (Harwell, Posey & Alphin, 2011), while increased three-dimensional complexity is associated with high abundance and diversity within a reef (Auster, Freiwald & Roberts, 2005; Wilson, Graham & Polunin, 2007). Our coral habitats represent a range in physical sizes, with *L. pertusa* creating the largest habitats, *M. oculata* intermediate sizes, and octocorals the smallest habitats; however, it is important to note that each coral type included a range in individual habitat sizes. Demopoulos, Bourque & Frometa (2014) previously suggested patch size as distinguishing community differences among *L. pertusa* habitats. *Lophelia pertusa* builds large structures that continually build upon themselves, expanding both horizontally and vertically over time, thus influencing hydrodynamic flow over large areas, promoting sediment accumulation both into and adjacent to the reef structure (Buhl-Mortensen et al., 2010). In addition, the long-term growth and senescence of a reef, with branches simultaneously accumulating new polyps and breaking down, supplies coarse-grained material to adjacent sediments with reef size influencing the total amount that accumulates. In contrast, the intermediate sizes of *M. oculata* colonies in the GOM are prone to fragmentation, also providing coarse grained material to the sediment pool as exhibited in our results. While the smaller size of *M. oculata* as compared to *L. pertusa* colonies may have a more tempered effect on local hydrodynamics, they still promote particle accumulation in adjacent sediments. Octocorals, in contrast to the scleractinian corals, can bend in response to currents, often orienting themselves perpendicular to the dominant flow (Mortensen & Buhl-Mortensen, 2005), and do not fragment in the same way. However, the size of the colonies can be comparable to those of *M. oculata,* and thus octocorals may influence hydrodynamic flows similarly to *M. oculata* colonies. While quantitative measurements of coral patch size were not measured, our results suggest that the amount of habitat heterogeneity influences adjacent infaunal communities, which has been observed in other types of deep-sea habitats (e.g., seeps (Cordes et al., 2010; Bourque et al., 2017), sponges (Raes & Vanreusel, 2005), and sedimented vents (Bell et al., 2016)). Additional sampling combined with detailed three-dimensional habitat mapping would be required to quantitatively define how individual coral habitats affect adjacent sediments communities.

While near-coral sediments at *L. pertusa* sites differed from background (>100 m) sediments (this study; Demopoulos, Bourque & Frometa, 2014), community differences between background and octocoral (>14 m) or *M. oculata* (>1,200 m) habitats were less distinct. Although it was not possible to statistically compare *M. oculata* near-coral and background communities due to the small sample size, the high R value suggests that the communities differ when compared to the significant results at *L. pertusa* site VK826, which had similar distances between near-coral and background communities (1,032–1,338 m). In contrast, background sediments associated with octocoral habitats were not significantly different from near-coral communities at distances 14–18 m away, less than the previously known minimum distance of 100 m for distinct background communities at *L. pertusa* site VK906 (Demopoulos, Bourque & Frometa, 2014). Demopoulos, Bourque & Frometa (2014) suggested that community turnover occurs at some distance less than 100 m from *L. pertusa* habitat given the difference in near-coral versus background communities. Combined with the results from the octocoral site, our results suggest that community turnover is occurring at some distance between 14 and 100 m away from coral habitats. In addition, among site community differences demonstrated in this study are consistent with previous results from deep-sea coral habitats in the GOM (Demopoulos, Bourque & Frometa, 2014; Demopoulos et al., 2016) and may be a function of local dynamics occurring near coral and background sediment environments. The distinct difference in both infaunal communities and functional trait composition within background sediments among coral habitats reflects the varying environments encompassed by each coral type, and further sampling of background sediments at both *M.  oculata* and octocoral habitats would provide additional information on the environmental drivers of these communities. Although distinct from near-coral communities, background communities at *L. pertusa* habitats were still unique from nearby GOM soft-sediment communities (Rowe & Kennicutt II, 2009; Demopoulos, Bourque & Frometa, 2014). Combined with the high similarity between near-coral and background sediments for *M. oculata* and octocoral habitats, our results suggest a sphere of influence ranging from 14 to 100m for all deep-sea coral habitats in the GOM.

Coral-associated infaunal communities exhibited differences from the general soft-sediment environments that dominate the northern Gulf of Mexico, consistent with previous studies (Demopoulos, Bourque & Frometa, 2014; Fisher et al., 2014). All three of the coral habitats contained macrofaunal densities in excess of the highest densities reported in the Deep Gulf of Mexico Benthos (DeGOMB) study near the head of the Mississippi Canyon (21,663 ind m^−2^, depth = 482–676 m; (Rowe & Kennicutt II, 2009). Only five of the 122 cores analyzed here, all of which were near corals, were below the second highest densities (6,000 ind m^−2^) reported from DeGOMB (Rowe & Kennicutt II, 2009) indicating higher densities within coral background sediments as well. Overall community composition also differed between DeGOMB and our study, with GOM soft-sediments containing lower proportions of polychaetes (47.2%) and higher proportions of amphipods (25.8%) than any of our coral habitats (Rowe & Kennicutt II, 2009). Near-coral communities also did not reflect the large-scale patterns in density and diversity present in northern GOM soft-sediment environments. Soft-sediment habitats in the northern GOM exhibit an exponential decline in density with depth (Rowe & Kennicutt II, 2009). In contrast, the quadratic relationship between density and depth for coral-adjacent sediments exhibited a mid-depth maximum between 600 and 800 m. While it appears that the typical depth-density pattern present in soft sediments is decoupled at coral habitats, the low *R*^2^ value of the quadratic relationship suggests there was a high amount of unexplained variation in this estimate, likely due to the patchy nature of the environment. Although diversity metrics used between the studies are not directly comparable due to differences in level of identification, diversity near coral habitats also exhibited a different pattern than the one established for the northern GOM. Diversity in the northern GOM has a parabolic relationship with depth, with maximum diversity between 1,100–1,300 m; specifically, within the depth range that we sampled at (263–1,095 m), diversity in DeGOMB sediment samples increased. However, diversity adjacent to deep-sea coral sediments exhibited no relationship to depth, further suggesting a localized influence of the habitat heterogeneity from coral habitats on supporting biodiverse sediment communities.

Although depth patterns with density and diversity differed from the overall northern GOM, depth individually explained the most variation in community structure, with further influence of grain size and food availability. As POC is known to decrease with distance from the coast and with depth in the GOM (Biggs, Hu & Muller-Karger, 2008), the overall food availability in a given area will be linked to the source amount. Although the corals may locally enhance organic carbon content, communities will always be limited by supply. Geographic location did not play a significant role in distinguishing coral infaunal community assemblages, suggesting that there are similarities among these communities regardless of whether they are in the eastern or western part of the northern GOM. In contrast, for functional traits only *L. pertusa* habitats were distinct, and longitude explained the most variation in functional trait composition across all habitats. Except for MC751, the *L. pertusa* sites are located northeast of most of the coral sites (Fig. 2). The distinct functional trait composition of *L. pertusa* habitats in combination with their location suggests there are additional site-specific environmental conditions not measured here (e.g., topography, current regimes, organic input) influencing the functional composition, including higher POC flux in the eastern GOM than the western GOM (Biggs, Hu & Muller-Karger, 2008) and known high current flows (Mienis et al., 2012). Additional sampling of geochemical variables at all coral locations and at *L. pertusa* habitats further west in the GOM will improve comparisons of the functional ecology of deep-sea coral infaunal communities across habitat types.

Although deep-sea corals are present worldwide, few investigations of infaunal communities have been performed (Henry & Roberts, 2007; Bongiorni et al., 2010; Demopoulos, Bourque & Frometa, 2014; Fisher et al., 2014; Demopoulos et al., 2016), despite growing evidence that associated sediments are unique. The three different coral types investigated here, *L. pertusa, M. oculata*, and octocorals, supported distinct infaunal communities in adjacent sediments, and each exhibits a sphere of influence that extends away from the coral habitat. With recent increased focus on conservation and management of these unique habitats, our results provide essential baseline information defining what constitutes a coral habitat for resource managers. These communities are influenced on small, local-scales by environmental controls such as organic carbon content and sediment grain size, both of which are likely influenced by the amount of habitat complexity exhibited by an individual coral habitat. Coral-associated communities are also influenced by large-scale controls, including depth and geographic location, suggesting that community differences may be region-specific. Our results provide the groundwork needed to address questions of infaunal community similarity and connectivity across additional coral habitats within the GOM and similar coral habitats worldwide. As deep-sea benthic biodiversity is linked to ecosystem functioning (Danovaro et al., 2008), our results provide important baseline information of how corals and their adjacent environments are structured and function to support diverse communities, increasing our understanding of overall coral ecosystem health.

##  Supplemental Information

10.7717/peerj.5276/supp-1 Supplemental Information 1 R code for univariate statistics Code for use with the program R to perform the univariate statistics included in the Results section.

10.7717/peerj.5276/supp-2 Supplemental Information 2 Whole core density and diversity metrics for cores collected adjacent to deep-sea coral habitats Each data point is a compilation of individual vertical fraction data where density represent the scaled sum of individuals and diversity metrics on whole core totals.

10.7717/peerj.5276/supp-3 Supplemental Information 3 Sediment geochemistry of cores collected near deep-sea coral habitats Each data point is a compilation of individual vertical fraction data values are averages. Blank cells indicate no data.

## Additional Information and Declarations

### Data Availability

The following information was supplied regarding data availability: ScienceBase: 10.5066/F7TH8KXD.
